# The Impact of Aboveground *Epichloë* Endophytic Fungi on the Rhizosphere Microbial Functions of the Host *Melica transsilvanica*

**DOI:** 10.3390/microorganisms12050956

**Published:** 2024-05-08

**Authors:** Chuanzhe Wang, Chong Shi, Wei Huang, Mengmeng Zhang, Jiakun He

**Affiliations:** College of Resources and Environment, Xinjiang Agricultural University, Urumqi 830052, China; 320223547@xjau.edu.cn (C.W.); huangwei@xjau.edu.cn (W.H.); 320223517@xjau.edu.cn (M.Z.); 320233612@xjau.edu.cn (J.H.)

**Keywords:** symbiosis, *Epichloë*, *Melica transsilvanica*, rhizosphere microbial functions, metagenomics

## Abstract

In nature, the symbiotic relationship between plants and microorganisms is crucial for ecosystem balance and plant growth. This study investigates the impact of *Epichloë* endophytic fungi, which are exclusively present aboveground, on the rhizosphere microbial functions of the host *Melica transsilvanica*. Using metagenomic methods, we analyzed the differences in microbial functional groups and functional genes in the rhizosphere soil between symbiotic (EI) and non-symbiotic (EF) plants. The results reveal that the presence of *Epichloë* altered the community structure of carbon and nitrogen cycling-related microbial populations in the host’s rhizosphere, significantly increasing the abundance of the genes (*por*A, *por*G, IDH1) involved in the rTCA cycle of the carbon fixation pathway, as well as the abundance of *nxr*AB genes related to nitrification in the nitrogen-cycling pathway. Furthermore, the presence of *Epichloë* reduces the enrichment of virulence factors in the host rhizosphere microbiome, while significantly increasing the accumulation of resistance genes against heavy metals such as Zn, Sb, and Pb. This study provides new insights into the interactions among endophytic fungi, host plants, and rhizosphere microorganisms, and offers potential applications for utilizing endophytic fungi resources to improve plant growth and soil health.

## 1. Introduction

Symbiosis between plants and microbes is a prevalent phenomenon in nature [1]. Microorganisms such as endophytes and rhizosphere and phyllosphere are involved in various stages of plant growth and development [2]. The symbiotic microbiota with plants can enhance the metabolic capacity of plants, improve the efficiency of nutrient uptake, and explore new nutritional pathways and defense processes for host plants [3,4,5]. In particular, rhizosphere microorganisms of plants are known as the second genome of plants because of their rich diversity, complex regulatory networks, and important roles in affecting plant growth and development and improving plant stress resistance [6]. Many studies have focused on the influence of environmental factors or pathogenic microorganisms on rhizosphere microorganisms [7]. Will symbiotic microorganisms, such as some endophytic fungi that are only present in the above-ground part of plants, have an impact on rhizosphere microorganisms in the subsurface part, and what impact will they have? Such research can help us better understand the interaction between endophytic fungi, host plants and rhizosphere microorganisms, which is very valuable in ecological research.

The endophytic fungi *Epichloë*, studied in this research, typically forms mutualistic symbiotic associations predominantly with cool-season grasses [8]. The host grass provides nutrients to *Epichloë*, which in turn enhances the host’s resistance to biotic and abiotic stresses such as drought, herbivory, and pathogen infections [9,10,11]. What sets it apart is that, due to long-term coevolution with the host, this symbiotic relationship is highly stable, allowing *Epichloë* to complete part or all of its lifecycle inside the host grass and eventually vertically transmit through seeds [12]. The unique and stable symbiosis of *Epichloë*, which confers beneficial traits to the host grass, has garnered widespread scientific interest and sparked a surge of research in microbiology, genetics, plant physiology, and animal science. An aspect often overlooked is its tissue-specific distribution within the host, typically found only in aboveground parts such as stems, leaf sheaths, seed coats, and endosperm layers [13,14]. The host plant can also coexist with both endophytic fungi and rhizospheric microorganisms. Due to its vertical transmission through seeds and its distribution restricted to aboveground tissues, *Epichloë* has temporal and spatial priority over rhizospheric microorganisms compared to those associated with the host’s roots [15,16]. In recent years, with the development and application of high-throughput sequencing technologies, it has been discovered that *Epichloë* fungi can influence the diversity of fungi and bacteria in the rhizosphere of grasses. For instance, endophytic fungi (*E. festucae* var. lolii strains AR1 and AR37) can alter the fungi and bacterial community structure of perennial ryegrass (*Lolium perenne* cv. Samson 11104) [17]. Casas et al. found that the endophytic fungi *E. occultans* altered the bacterial community composition of potted perennial ryegrass soil but had no significant impact on the fungi community [18]. Endophytic fungi *E. coenophiala* significantly affected the fungi community in the rhizosphere of tall fescue and increased the relative abundance of certain microbial genera [19]. This indicates that aboveground *Epichloë* endophytic fungi can impact rhizospheric microorganisms. However, studies have mainly focused on artificial grasses such as tall fescue and perennial ryegrass, and have only elucidated the effects of *Epichloë* on the structure and diversity of host rhizospheric microbial communities, while the analysis of its influence on rhizospheric microbial functional genes and functional groups is relatively lacking.

But there are also plenty of *Epichloë* fungi in native grasslands. *Melica transsilvanica*, the host of this study, is a perennial grass belonging to the gramineous family. It coexists symbiotically with *E. guerinii* [20,21]. It is extensively dispersed throughout Europe, Central Asia, Russia, Siberia, and the Caucasus; nevertheless, it is only found naturally in China, on the north slope of the Tianshan Mountain, where it is highly valuable for study due to its good palatability and soft grass [22]. As experimental technology advances, sequencing depth is growing and the metagenomic study of soil microbial function is being utilized progressively [23]. In this study, metagenomics was used to sequence the rhizosphere soil of *M. transsilvanica*-*E. guerinii* symbionts (EI) and non-symbionts (EF). This study aims to achieve the following: (1) to further analyze the differences in the functional genes of rhizosphere soil microorganisms, focusing on functional genes related to soil nutrient cycling, virulence factors and heavy metal resistance; and (2) to analyze the differences in the functional groups of microorganisms carrying functional genes. In order to improve the understanding of *Epichloë*—host plant—rhizosphere microbial interaction, it also provides a theoretical basis for better use of endophytic fungi resources, improve agricultural production efficiency, and promote soil health and ecological sustainable development.

## 2. Materials and Methods

### 2.1. Test Materials and Sample Collection

The mountain steppe on the north slope of the Tianshan Mountain, China (87.42° E, 43.47° N, elevation 1393 m), with an annual mean temperature of 3 °C and an annual precipitation of 208.4 mm, is where the *Melica transsilvanica* seeds were harvested in August 2020. To properly destroy the endophytic fungi in the seeds, one portion was kept at 4 °C in a refrigerator, and the other portion was exposed to dry heat at 70 °C for 25 days [24]. The treated seeds were propagated in August 2022 in order to prevent the effects of dry heat on the seeds themselves. Aniline blue staining was used to confirm that the propagated seeds had no bacteria present, and they were then seeded and proved to be non-symbiontes (EF) in further testing [25]. After being sown into sterilized vermiculite in February 2023, seeds that had not been microwave-sterilized developed into three or four leaves. The plants’ sheaves of leaves were ripped, then aniline blue was added. Plants exhibiting endophytic fungi mycelia were found to be symbionts (EI).

To minimize external environmental influences, we conducted indoor potted plant experiments. Soil from the northern slope of the Tianshan Mountains (87.42° E, 43.47° N, 1393 m above sea level) was mixed with sterile vermiculite in a 3:1 volume ratio and placed into 1 L nutrient bowls. Each treatment, EI and EF, had 18 replicate bowls, totaling 36. Each bowl was planted with 5 plants of consistent growth from the sterile vermiculite substrate. Daily additions of deionized water maintained a 70% soil moisture content, and any fallen leaves or other plants were promptly removed. After 8 months of growth in a controlled environment chamber, we collected rhizosphere soil. Large pieces of soil without roots were gently shaken off, and soil adhering to fine roots was brushed off with a sterile brush. The soil was quickly passed through a 2 mm sieve to remove roots, debris, and other impurities, then stored at −80 °C for metagenomic sequencing [26]. For each treatment, rhizosphere soil from 30 plants in a total of 6 nutrient bowls was randomly mixed to create one sample, with 3 samples prepared for each group.

### 2.2. Soil Metagenomics Sequencing

The PowerSoil DNA Isolation Kit from Mobio Laboratories was used to extract DNA from a 0.25 g rhizosphere soil sample in accordance with the manufacturer’s instructions. With the use of a Thermo Fisher Scientific NanoDrop 1000 Spectrophotometer (Waltham, MA, USA), the extracted DNA’s quality and amount were evaluated. Wekemo Tech Group Co., Ltd. (Shenzhen, China) employed the Illumina NovaSeq 6000 (Illumina, San Diego, CA, USA) platform for library preparation and sequencing, adhering to the manufacturer’s guidelines. After sonication to reach a 350 bp size range, end repair, adenylation, and amplification were performed on the genomic DNA according to the Illumina sequencing methodology. Using the Trimmomatic-based KneadData program (v0.10.0), quality control was carried out. The efficacy and efficiency of the quality control procedure were assessed using FastQC (v0.11.9). After removing adaptor sequences and reducing low-quality reads from the initial metagenomic sequencing data—which contained reads with N bases and a minimum quality score cutoff of 20—clean reads (Table S1) were obtained.

### 2.3. Metagenomic Data Processing

Functional database annotation: Initiating from the reads of quality-controlled genomic data, the HUMAnN3 software (v3.0.2), which is based on the DIAMOND algorithm, was utilized to map the reads from each sample against the KEGG gene database. The abundance of functional categories was calculated based on the sum of gene abundances corresponding to KO (Kyoto Encyclopedia of Genes and Genomes) and Pathway terms. For further annotation related to virulence factors and heavy metal resistance, the DIMOND software (v 0.8.17.79) was employed, leveraging the Virulence Factors of Bacterial Proteins Database (VFDB) and the BacMet database, which are specialized repositories for such annotations. Functional species annotation: The taxonomic composition of the samples was determined by aligning the sequences against a proprietary microbial nucleic acid database constructed by Microeco Ltd., which includes sequences from bacteria, fungi, archaea, and viruses that are present in the NCBI NT nucleic acid database and the RefSeq complete genome database. Subsequently, the Bracken software (v2.7) was used to estimate the actual abundance of species within the samples, thereby providing a comprehensive profile of both functional genes and their host taxa abundance information. This integrated approach ensures a thorough understanding of the functional capabilities and species diversity within the microbial communities under study.

### 2.4. Data Visualization and Statistical Analysis

By utilizing the STAMP (Version 2.1.3) software, we conducted a differential analysis of the abundance of functional genes between groups. Furthermore, we employed the Bioinformatics Cloud platform (Wekemo Bioincloud: https://www.bioincloud.tech/task-meta accessed on 4 May 2024) to perform a principal component analysis (PCA) on the functional gene sets between the two groups. To assess the variability in microbial functional communities among different group samples, we implemented a non-metric multidimensional scaling (NMDS) analysis based on the Bray–Curtis distance metric, followed by a linear discriminant analysis (LDA) [27].

## 3. Results

### 3.1. C Cycling

**Figure 1:**
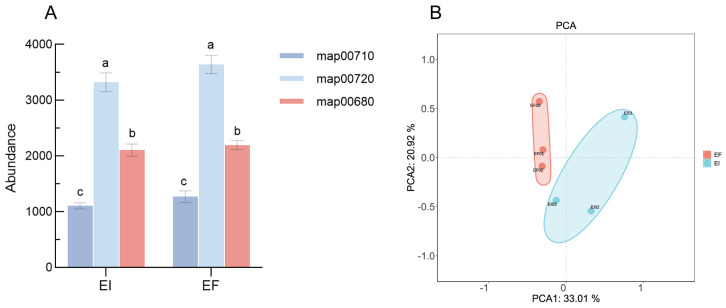
Total abundance of gene sets in the three main pathways of the carbon cycle (**A**); different lowercase letters above the bars indicate significant differences, as determined by one-way ANOVA (*p* < 0.05) and the principal component analysis (PCA) of carbon cycle gene sets (**B**).

**Figure 2:**
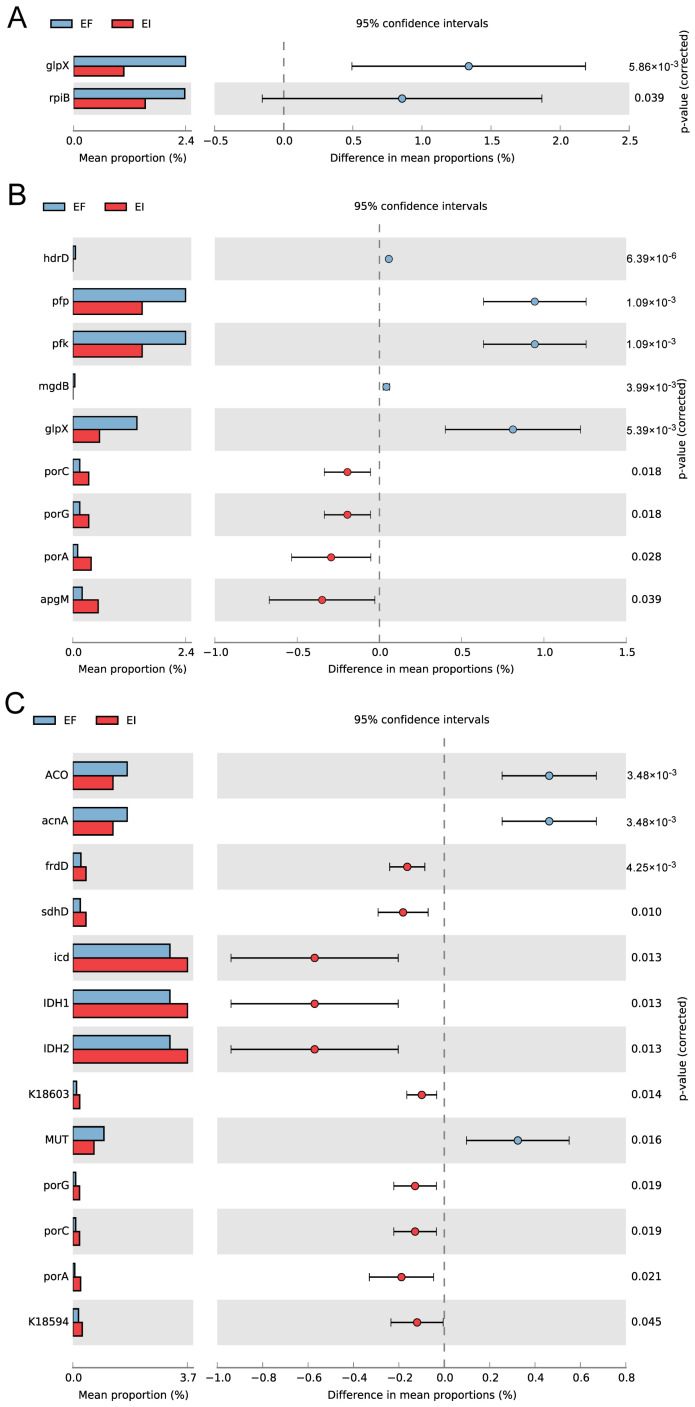
Intergroup variations in pathway gene sets for map00710 (**A**), map00680 (**B**), and map00720 (**C**) analysis.

Within the KEGG database, the two carbon fixation pathways are delineated by the functional pathways map00710 (carbon fixation in photosynthetic organisms) and map00720 (carbon fixation in prokaryotes), while map00680 is associated with methane metabolism. No significant differences were observed in the overall abundance of genes related to the three pathways between the EI and EF groups (Figure 1A). However, the principal component analysis (PCA) results for gene sets related to the carbon cycle revealed a clear separation between the two groups along the PC1 and PC2 axes, indicating a divergence in their functional gene compositions. Further analysis using STAMP indicated (Figure 2) that the abundance of *glp*X and *rpl*B genes, which are part of the carbon fixation pathway in photosynthetic organisms, was significantly higher in the EF group (*p* < 0.05). Conversely, in the carbon fixation pathway of prokaryotes, the genes encoding isocitrate dehydrogenase (icd and IDH) were not only more abundant in the EI group but also significantly higher than in the EF group (*p* < 0.05). Notably, the *por*A, *por*C, and *por*G genes, which encode key enzymes of the reductive tricarboxylic acid cycle (rTCA cycle), pyruvate ferredoxin oxidoreductase (PFOR), were present at lower abundances in the EI group but were significantly higher than in the EF group (*p* < 0.05). This pathway, proposed by Evans in 1966 [28], is one of the few autotrophic CO_2_ fixation pathways in bacteria, suggesting a stronger prokaryotic carbon fixation potential in the EI rhizosphere.

**Figure 3:**
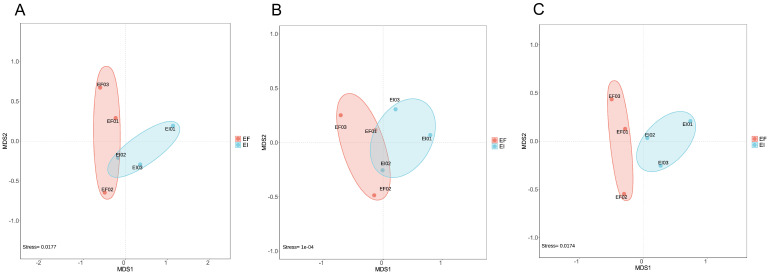
Nonmetric multidimensional scaling (NMDS) analysis of functional class groups by map00710 (**A**), map00720 (**B**), and map00680 (**C**).

**Figure 4:**
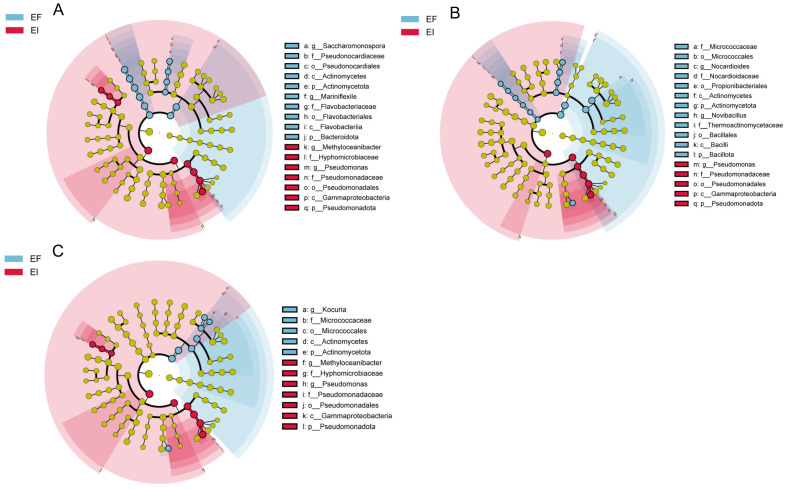
Variations in microorganisms’ lefse analyses in the routes map00710 (**A**), map00720 (**B**), and map00680 (**C**) (LDA > 2).

We traced the microbial origins of the genes within the aforementioned pathways. Figure 3 presents the non-metric multidimensional scaling (NMDS) analysis of the three functional communities, all of which exhibit good representativeness (stress < 0.05). The results demonstrate that the community structures of the three functional groups in both the EI and EF groups are distinct. Further analysis using LEfSe to identify functionally enriched microbes with significantly altered abundance in the rhizosphere soil revealed (Figure 4) that the *Pseudomonas* genus exhibited significant differences at the genus level across all three functional groups (LDA > 2). As a typical microorganism of the reductive acetyl-CoA carbon fixation pathway [29], the higher relative abundance of *Pseudomonas* in the EI rhizosphere soil suggests a greater potential for CO_2_ fixation.

### 3.2. N Cycling

**Figure 5:**
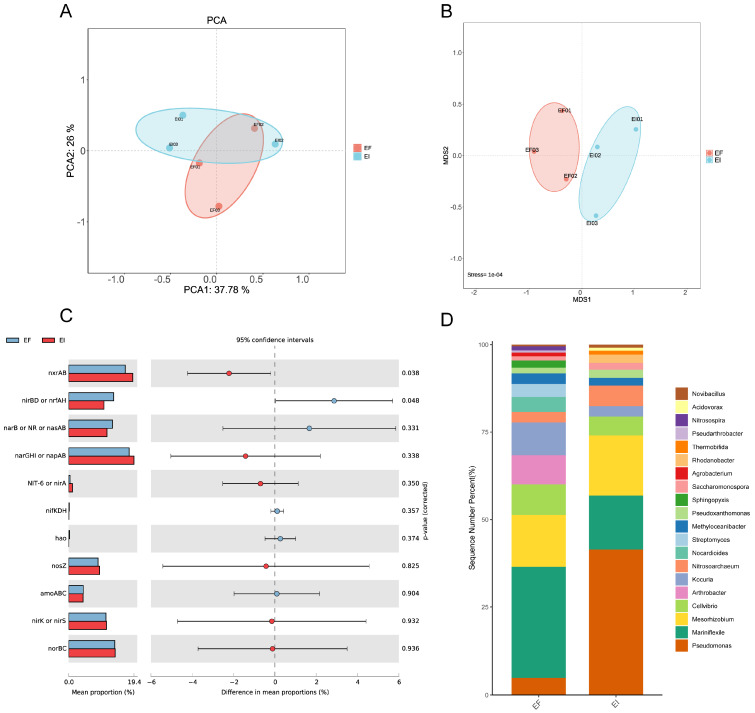
Principal component analysis (PCA) of the gene set associated with the nitrogen cycle (map00910) (**A**), differential gene abundance in major pathways (**C**), and the taxonomic origins of functional groups related to the nitrogen cycle (**D**) alongside a non-metric multidimensional scaling (NMDS) analysis (**B**).

Our principal component analysis (PCA) of the gene set of the (map00910) nitrogen cycle pathway showed that the distance between EI and EF samples was small (Figure 5A). Subsequently, we mapped the relevant genes to specific nitrogen cycle pathways (Table S2) and conducted a STAMP analysis (Figure 5C). The results indicated that the abundance of the *nxr*AB gene was significantly higher in the EI group compared to the EF group (*p* < 0.05). The *nxr*AB gene primarily participates in the nitrification process, converting nitrite to nitrate. In contrast, the abundance of *nir*BD and *nrf*AH was significantly higher in the EF group than in the EI group (*p* < 0.05), corresponding to the pathway of dissimilatory nitrate reduction to ammonia. Therefore, it can be inferred that the rhizosphere microbiota of EI plants exhibit a stronger nitrification capacity, which promotes the host’s nitrogen uptake. We also traced the microbial origins of the genes within the nitrogen cycle pathways (Figure 5D). *Pseudomonas* was found to be more enriched in the rhizosphere of EI, and *Nitrosoarchaeum*, as an ammonia-oxidizing archaeon, also showed a higher abundance in EI, which may be key to the stronger nitrification capacity in the EI rhizosphere. Furthermore, NMDS analysis revealed differences in the community composition of nitrogen cycle-related functional groups between EI and EF (Figure 5B).

### 3.3. P Metabolism

**Figure 6:**
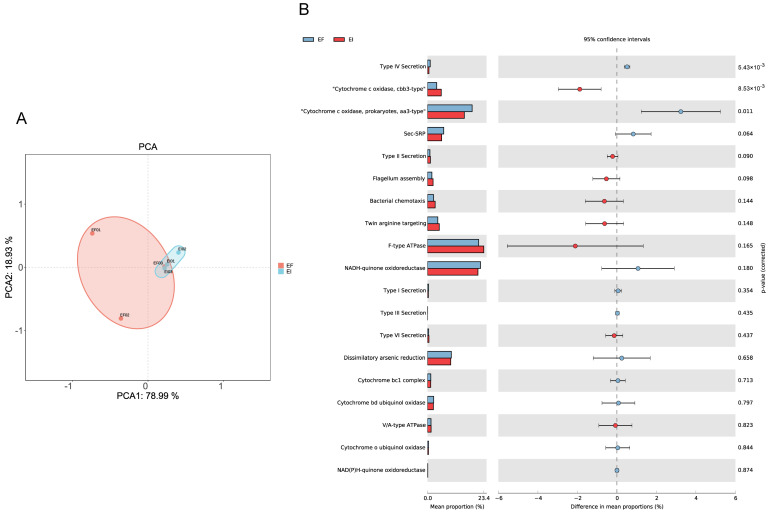
Principal component analysis (PCA) of the gene set associated with phosphorus metabolism (**A**), and the variation in gene abundance within key pathways (**B**).

We separately annotated the abundance of genes related to phosphorus metabolism. PCA analysis showed clear separation and differences between the EF and EI groups in PC1 and PC2, indicating significant differences in the genes related to phosphorus metabolism between the two (Figure 6A). Classifying these functional genes into relevant pathways, it appears that the rhizosphere microbes of EF plants are more capable of promoting phosphorus cycling. Specifically, genes related to Sec-SRP (Secreted Soluble Reactive Phosphorus) involved in phosphorus transformation and cycling were significantly more abundant in EF than in EI (Figure 6B).

### 3.4. Resistance to Heavy Metals and Virulence Factors

**Figure 7:**
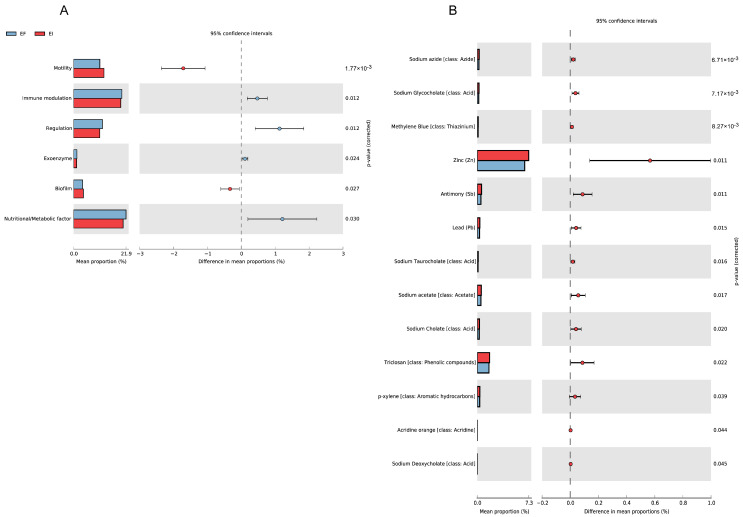
Variations in the rhizosphere microorganisms of EI and EF plants with respect to the virulence factor (**A**) and heavy metal resistance (**B**).

Through annotation with the VFDB (virulence factor) database, we found that the abundance of virulence factors in the rhizosphere of EI plants was lower, and the types were fewer compared to EF plants (Figure 7A). This indicates that the presence of the Epichloë endophytic fungi can reduce the enrichment of virulence factors in the host rhizosphere, thereby enhancing host resistance. Annotation with the BacMet (heavy metal resistance) database revealed that the rhizosphere of EI plants was enriched with more microbial functional genes resistant to Zn, Sb, and Pb (Figure 7B).

## 4. Discussion

In terms of carbon cycling, previous studies have shown that the host’s root exudates and litter are the main sources of soil organic carbon. The infection of endophytic fungi increases the host’s biomass, so that the litter into the soil increases, resulting in an increase in soil organic carbon and other substances [30]. The presence of endophytic fungi may also promote the secretion of some compounds by the host root, directly affecting the content of soil carbon [31]. Through the analysis of functional genes and related microbial groups in rhizosphere soil, this study found that the presence of *Epichloë* increased the abundance of the key gene *por*A in the host rhizosphere microorganism rTCA, and increased the abundance of Pseudomonas in the corresponding functional groups. Studies have shown that PFOR encoded by *por*A is an important enzyme in the rTCA cycle, which is involved in the transformation of pyruvate to acetyl coenzyme A. *Pseudomonas* is a typical reducing acetyl coenzyme A carbon sequestration pathway [32,33]. Yang et al. [34] found, in the carbon sequestration study of soil microorganisms in arid desert oases, that the rTCA cycle indicated by the difference in the *por*A gene is one of the most common carbon sequestration pathways. When Ren et al. [35] used the macrogenome combined with the macrotranscriptome to study the soil microbial function in Tarim Basin, he also found that the rTCA cycle driven by *por*A, *por*G and IDH1 genes is one of the carbon sequestration pathways. These two aspects indicate that the presence of *Epichloë* can enrich the host rhizosphere with more microorganisms with the ability to fix CO_2_, and also increase the abundance of carbon-fixing function genes, which may be the third reason why endophytic fungi increase the organic carbon content of the host rhizosphere soil.

In terms of nitrogen cycling, endophytic fungi can reduce the impact of low nitrogen stress on the host by directly enhancing the absorption of nutrients by the host. There are also related studies that evaluate the nitrification potential of soil by measuring the nitrate nitrogen in soil; find that the presence of endophytic fungi can increase soil nitrification [36]. Endophytic fungi infection can reduce the activity of nitrification-inhibiting substances in the host rhizosphere, and then affect soil nitrification [37], which is consistent with the conclusion that the rhizosphere microorganisms of EI plants have stronger nitrification in this study. For the functional gene level of *Epichloë* affecting the nitrogen cycling of host rhizosphere microorganisms, only two articles have reported that the presence of *Epichloë festucae* affects the nitrogen cycling process of rhizosphere microorganisms of host ryegrass; under low nitrogen stress, the abundance of ammonia-oxidizing bacteria and ammonia-oxidizing archaea in the rhizosphere of the symbiotic species increased. And there are differences in the functional groups of nitrogen-fixing microorganisms between the symbiotic and non-symbiotic species [38,39]. The results of this study were more prominent than those of our study, but in the same direction as our conclusions, which may be due to the fact that the above experiments were conducted under low nitrogen conditions, and the role of endophytic fungi was highlighted.

Interestingly, the results of the comprehensive functional groups showed that *Epichloë* could recruit a large number of growth-promoting bacteria *Pseudomonas* in the host rhizosphere, which was consistent with the conclusions of previous studies, that is, *Pseudomonas* bacteria were the dominant bacterial groups widely distributed in the rhizosphere, seed and leaf of *Epichloë*–*Achnatherum inebrians* symbiosis [40]. *Pseudomonas* had a nitrogen fixation effect in the plant rhizosphere, as well as the role of regulating plant hormones and increasing abiotic stress tolerance [41]. As early as 1996, it was found that *Pseudomonas* was a strong candidate soil inoculant bacteria to improve crop yield [42]. Recent studies also found that inoculation of *P. aeruginosa* could promote the growth of pea (*Pisum sativum*) [43], and inoculation of *P. moraviensis* could positively promote the growth and physiology of wheat (*Triticum aestivum*), while significantly improving the soil organic matter and nitrogen availability [44]. Therefore, *Pseudomonas* play an important role in promoting plant growth and development. We concluded that the overall growth-promoting effect of *Epichloë* on the host was partially attributed to the recruitment of *Pseudomonas* by symbiosis. As for why endophytic fungi chose it as a “helper”, it is also a scientific question worthy of future research.

In the context of phosphorus metabolism, this study did not observe significant differences between the EI and EF groups, and it is even possible that the potential of rhizosphere microorganisms in phosphorus metabolism for EI plants is lower than that for EF. However, some research has indicated that *E. gansuensis* improves the growth of its host *A. inebrians* under low phosphorus stress by regulating amino acid metabolic pathways, amino acid content, and organic acid content, and by increasing phosphorus utilization efficiency [45]. This phenomenon may be related to the symbiotic relationship between plants and endophytic fungi, where the endophytic fungi may enhance the host’s own phosphorus absorption and reduce the host’s reliance on rhizosphere microorganisms with the function of activating phosphorus or secreting organic phosphorus. Previous studies have concluded that *Epichloë* has no significant effect on the content of the available phosphorus in host rhizosphere soil under non-stress conditions, which can also confirm this view [46].

The influence of *Epichloë* on the host in producing specific metabolites to resist pathogen invasion in both aboveground and belowground parts has been extensively studied [47]. In this study, the lower abundance and diversity of virulence factors enriched in the root zone of EI plants further corroborate this finding. Additionally, *Epichloë* can enhance host tolerance to heavy metal ions such as cadmium (Cd^2+^), aluminum (Al^3+^), zinc (Zn^2+^), and copper (Cu^2+^) in soil. According to a large number of previous studies, under the stress of toxic heavy metals, symbiotic plants have higher antioxidant enzyme activity and chlorophyll a and b contents, and lower proline and malondialdehyde contents than non-symbiotic plants, indicating that the presence of *Epichloë* can better activate the antioxidant system of host plants [48,49,50]. The activity of antioxidant enzymes in plants was increased to reduce the ROSs (reactive oxygen species) produced under heavy metal stress. The oxidative damage of ROSs to nucleic acid, membrane and chloroplast pigment was alleviated. In addition to protecting photosynthetic pigments from destruction, *Epichloë* also enhances the activity of the enzymes related to photosynthesis and chlorophyll synthesis [51]. Other studies have shown that heavy metal stress, especially Cu^2+^, can cause plants to produce excessive ethylene and accelerate plant senescence, while *Epichloë* can regulate ethylene synthesis and inhibit plant senescence, thereby enhancing the host’s tolerance to heavy metals [52]. In conclusion, endophytic fungi enhance the host’s tolerance to heavy metal ions by improving the host’s antioxidant capacity and photosynthesis level, and by activating related hormone signals. In this study, it was found that the abundance of the functional genes of microorganisms resistant to Zn, Sb and Pb in the rhizosphere microorganisms of EI plants was higher than that of EF plants. To a certain extent, this indicates that Epichloë enhances the host’s tolerance to heavy metal ions, which not only affects the physiological response of the host plant itself, but it also indirectly affects the resistance of rhizosphere microorganisms symbiosis with the host to heavy metals.

This study provides new insights into the interactions among *Epichloë*, host plants, and rhizosphere microorganisms, offering potential applications for utilizing these microbial relationships to improve plant growth and soil health. However, there are some limitations in the study. For example, although metagenomic approaches can reveal the functional gene composition of microbial communities, there is a lack of direct measurement of microbial activity and metabolic processes. Future research could further explore the complex interaction mechanisms between endophytic fungi and host plants by combining metagenomics, transcriptomics, and metabolomics, among other multi-omic approaches, to investigate how these interactions affect plant growth and the functional aspects of soil ecosystems.

## 5. Conclusions

The presence of *Epichloë* can change the community structure of the functional groups related to the carbon cycle in the rhizosphere microorganisms of the host Melica transsilvanica, and significantly increase the abundance of *por*A, *por*G and IDH1 genes in the rTCA cycle of the carbon sequestration pathway. This may be the third reason why endophytic fungi increase the organic carbon content of the host rhizosphere soil in addition to the other two main factors, namely, increasing host litter and altering host root exudates. The abundance of the *nxr*AB gene in the rhizosphere microbial nitrogen cycle pathway in EI plants was significantly higher than that in EF plants, and the community structure of related functional groups also changed. The changes in carbon and nitrogen nutrient cycling in rhizosphere microorganisms may be related to the fact that the presence of *Epichloë* recruits a large number of *Pseudomonas* bacteria in the host rhizosphere. In addition, the presence of *Epichloë* also reduces the enrichment of virulence factors by host rhizosphere microorganisms and significantly increases the accumulation of heavy metal resistance genes to Zn, Sb and Pb. To a certain extent, it indicates that *Epichloë* enhances the host’s resistance to soil-borne pathogens and heavy metal ions, which not only affects the physiological response of host plants, but also indirectly affects the rhizosphere microorganisms that are symbiotic with the host and cooperate with the host to develop resistance to stress.

## Data Availability

The sequences were submitted to the NCBI database (ID: PRJNA949598).

## Supplementary Materials

The following supporting information can be downloaded at: https://www.mdpi.com/article/10.3390/microorganisms12050956/s1, Table S1: Data output quality; Table S2: Key genes corresponding to pathways associated with the nitrogen cycle; Table S3: Key genes corresponding to pathways related to phosphorus metabolism.
