# Supplementary material for: The Impact of Aboveground Epichloë Endophytic Fungi on the Rhizosphere Microbial Functions of the Host Melica transsilvanica
Source: Microorganisms. 2024 May 8;12(5):956. doi: 10.3390/microorganisms12050956 (PMC11124418; doi:10.3390/microorganisms12050956)
Supplement: Supplementary file 1 [file microorganisms-12-00956-s001.zip › microorganisms-2993555-supplementary.pdf]

Table S1. Data output quality

Sample ID	Insert Size(bp)	SeqStrategy	RawReads(#)	Raw Base(GB)	%GC	Raw Q20(%)	Raw Q30(%)	Clean Reads(#)	Cleaned(%)	Clean Q20(%)	Clean Q30(%)
EF01	350	(150:150)	26795755	8.04	64	98.86	96.67	25743882	96.07	99.61	98.37
EF02	350	(150:150)	29191620	8.76	64	98.83	96.56	28028786	96.02	99.59	98.29
EF03	350	(150:150)	29670609	8.90	64	98.95	96.92	28667070	96.62	99.62	98.43
EI01	350	(150:150)	20625771	6.19	63	98.86	96.71	19860204	96.29	99.61	98.38
EI02	350	(150:150)	23635495	7.09	64	98.81	96.52	22571714	95.50	99.60	98.32
EI03	350	(150:150)	25990888	7.80	63	98.86	96.69	24987748	96.14	99.61	98.38

Table S2. Key genes corresponding to pathways associated with the nitrogen cycle

Pathway	Genes
Dissimilatory nitrate reduction, nitrate -> nitrite (narGHI or napAB)	K00370,K00371,K00374,K02567,K02568
Dissimilatory nitrate reduction, nitrite -> ammonia (nirBD or nrfAH)	K00362,K00363,K03385,K15876
Assimilatory nitrate reduction, nitrate -> nitrite (narB or NR or nasAB)	K00367,K10534,K00372,K00360
Assimilatory nitrate reduction, nitrite -> ammonia (NIT-6 or nirA)	K17877,K00366
Denitrification, nitrite -> nitric oxide (nirK or nirS)	K00368,K15864
Denitrification, nitric oxide -> nitrous oxide (norBC)	K04561,K02305
Denitrification, nitrous oxide -> nitrogen (nosZ)	K00376
Nitrogen fixation, nitrogen -> ammonia (nifKDH)	K02586,K02588,K02591,K22896,K22897,K22898,K22899
Nitrification, ammonia -> hydroxylamine (amoABC)	K10944,K10945,K10946
Nitrification, hydroxylamine -> nitrite (hao)	K10535
Nitrification, nitrite -> nitrate (nxrAB)	K00370,K00371

Table S3. Key genes corresponding to pathways related to phosphorus metabolism

**Pathway**	**Genes**
F-type ATPase	K02111,K02112,K02115,K02113,K02114,K02108,K02109,K02110
V/A-type ATPase	K02117,K02118,K02119,K02120,K02121,K02122,K02107,K02123,K02124
NADH-quinone oxidoreductase	K00330,K00331,K00332,K00333,K00331,K13378,K13380,K00334,K00335,K00336,K00337,K00338,K00339,K00340,K00341,K00342,K15863,K00343
NAD(P)H-quinone oxidoreductase	K05574,K05582,K05581,K05579,K05572,K05580,K05578,K05576,K05577,K05575,K05573
Cytochrome c oxidase, cbb3-type	K00404,K00405,K15862,K00406,K00407
Cytochrome bd ubiquinol oxidase	K00425,K00426,K00424,K22501
Cytochrome o ubiquinol oxidase	K02300,K02299,K02298,K02297
Cytochrome c oxidase, prokaryotes, aa3-type	K02275,K02274,K02276,K15408
Cytochrome bc1 complex	K00412,K00413,K00410,K00411,K00414,K00415,K00416,K00417,K00418,K00419,K00420
Type I Secretion	K12340,K11003,K11004
Type III Secretion	K03221,K04056,K04057,K04058,K04059,K03219,K03222,K03223,K03224,K03225,K03226,K03227,K03228,K03229,K03230
Type II Secretion	K02452,K02453,K02454,K02455,K02456,K02457,K02458,K02459,K02460,K02461,K02462,K02464,K02465
Type IV Secretion	K03194,K03197,K03198,K03200,K03202,K03204,K03201,K03203,K03195,K03199,K03196,K03205
Type VI Secretion	K11904,K11903,K11906,K11891,K11892,K11907,K11912,K11913,K11915
Sec-SRP	K03072,K03074,K12257,K03073,K03075,K03076,K03210,K03217,K03070,K13301,K03110,K03071,K03106
Twin arginine targeting	K03116,K03117,K03118,K03425
Bacterial chemotaxis	K03406,K05874,K05875,K05876,K05877,K03776,K10108,K10439,K10540,K12368,K03407,K03408,K03413,K03410,K03414,K03409,K03412,K13924,K03415,K03411,K00575,K02410,K02416,K02417,K02556,K02557
Flagellum assembly	K02402,K02403,K02398,K02405,K02406,K02407,K02397,K02396,K02414,K02389,K02390,K02391,K02392,K02393,K02394,K02387,K02388,K02408,K02409,K02410,K02416,K02417,K02400,K02401,K02411,K02412,K02418,K02419,K02420,K02421,K13820,K02556,K02557,K21217,K21218,K02399,K02413,K02422,K02423,K02386
Dissimilatory arsenic reduction	K00537,K03741,K18701,K03325,K03893,K03892,K01551
